# Single-Cell Gene Expression Analysis in Patients with Medullary Sponge Kidney and a Retrospective Study

**DOI:** 10.1155/2022/7688947

**Published:** 2022-11-11

**Authors:** Ming Li, Da-Ming Xu, Shu-Bin Lin, Zheng-Liang Yang, Teng-Yu Xu, Jin-Huan Yang, Jun Yin

**Affiliations:** ^1^Division of Urological Surgery, Second Affiliated Hospital of Shantou University Medical College, Shantou, Guangdong, China; ^2^Division of Hematology, Second Affiliated Hospital of Shantou University Medical College, Shantou, Guangdong, China; ^3^Department of Clinical Laboratory Medicine, Second Affiliated Hospital of Shantou University Medical College, Shantou, Guangdong, China

## Abstract

**Objective:**

To establish better diagnosis thinking and provide advanced understanding of MSK, the CT imaging features, clinical characteristics, and the expression of suspected genes in the kidney spatiotemporal immune zonation and fetal renal development were investigated.

**Methods:**

17 patients with MSK hospitalized in our hospital were selected as our research subjects. Human Phenotype Ontology, MalaCards: The Human Disease Database, GeneCards: The Human Gene Database, Human Protein Atlas, and Single Cell Expression Atlas were used to analyze this disease.

**Results:**

In our 17 patients, the incidence of MSK tended to be the same in male and female, and the onset age of MSK was probably 31-50 years old. The top one related disease of MSK was nephrocalcinosis and the most frequent phenotype related to MSK was nephrolithiasis. In addition, the expression of HNF1B, CLCN5, GDNF, ATP6V0A4, ATP6V1B1, LAMA2, RET, ACAN, and ABCC8 has been implicated in both human kidney immune zonation and fetal kidney development.

**Conclusions:**

HNF1B, CLCN5, GDNF, ATP6V0A4, ATP6V1B1, LAMA2, RET, ACAN, and ABCC8 could be independent indicators for the diagnosis and preventive intervention of MSK patients, and abnormal kidney development due to mutations in key genes was the underlying cause of MSK.

## 1. Introduction

Medullary sponge kidney (MSK) is a rare renal congenital tract malformation characterized by dilatation of the medullary collecting ducts, accompanied by kidney stones, renal cysts, urinary tract infection, hematuria, calcinosis, pyelonephritis, and renal tubular acidosis [1–3]. The renal anatomy of the patient with MSK revealed abnormal cystic dilated renal ducts in the medulla forming a cavernous appearance. The prevalence of MSK is uncertain, at about 1/5000 persons, due to the clinical presentation of MSK patients is usually indolent [4]. Notably, the detection rate of MSK can be increased to 20% in patients with recurrent kidney stones [5]. Although Gambaro et al. first reported MSK in 1938, the pathogenesis and clinical phenotype of MSK remain unclear to this day [6]. As MSK is a rare disease, there are few studies on MSK at present. The congenital disease involving abnormal epigenetic mechanism and the acquired disease secondary to calcium accumulation or hyperparathyroidism are the main explanations for the occurrence of MSK [6, 7]. Some scholars suggested that MSK was an autosomal dominant inherited disease but due to its sporadic nature, MSK has rarely been found to be inherited in the family via an autosomal dominant manner, which makes MSK rarer in infants [3, 6, 8]. The gold standard for MSK diagnosis is intravenous urography (IVU) which can reveal the pathognomonic images of dilated calyces and enlarged medulla collecting ducts [9]. However, in clinical work, the applying of ultrasound and computed tomography (CT) are more popular than IVU to find the urinary calculi, which increases the difficulty of MSK diagnosis [10]. Of note, combined with our clinical findings and existing literature reports, MSK was often associated with the occurrence of a variety of congenital abnormalities inside and outside the kidney, which suggested that MSK may be a systemic disease. Therefore, the pathogenesis of MSK should be explained by a new perspective. Tissue-lived immune cells are significant for organ homeostasis and defense, which can form a functional interaction network with renal epithelial cells to perform the functions [11]. In addition, human fetal renal development can make a comprehensive understanding of the possible developmental origins of kidney disorders [12].

Herein, we analyzed the patients with a diagnosis of MSK in our hospital over the past 10 years, investigated the CT imaging features and clinical characteristics, and preliminary described the expression of suspected genes in the kidney spatiotemporal immune zonation and fetal renal development through the single-cell gene expression, which help establish better diagnosis thinking and provide advanced understanding of MSK pathogenesis.

## 2. Materials and Methods

### 2.1. Patient Enrollment

17 patients hospitalized in the Second Affiliated Hospital of Shantou University Medical College between 2011-11-04 and 2021-11-04 were selected as our research subjects. Their clinical data were collected (Table 1). The human phenotype analyses of MSK were collected from the Human Phenotype Ontology (https://hpo.jax.org/app). The data of related diseases and related genes of MSK were collected from MalaCards (https://www.malacards.org). GeneCards (https://www.genecards.org) was applied for GO analysis (cellular components, biological processes, and molecular functions) of MSK (https://www.genecards.org). The data of protein expression location of genes in human cells were collected from the Human Protein Atlas (https://www.proteinatlas.org). Gene expression in spatiotemporal immune zonation of the human kidney and single cell RNA-sequencing of human fetal kidneys were analyzed by the Single Cell Expression Atlas (https://www.ebi.ac.uk/gxa/sc/home).

## 3. Results

### 3.1. Imaging Studies

**Figure 1:**
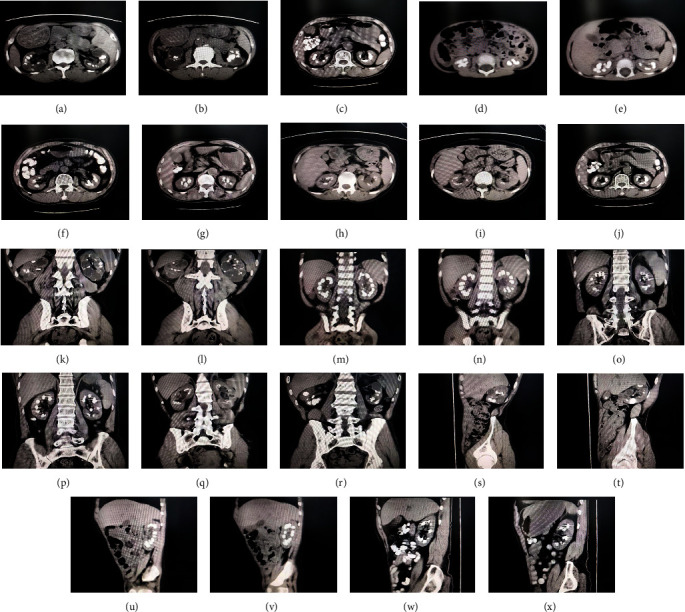
CT imaging features of MSK patients.

We reviewed 17 patients with MSK and found the following CT imaging features. In the CT imaging, the size, shape, and position of both kidneys were normal, the renal capsule was intact, and the surrounding fat space was clear. The dilated collecting tubes appear as streaks or sacs of low density. High density calculi shadows are often irregular and nodular, usually in the bilateral renal papillary areas, bilateral renal medulla, or bilateral renal calyces (Figure 1).

### 3.2. Clinical Data Analysis, Human Phenotypes Analysis and GO Analysis of MSK

**Figure 2:**
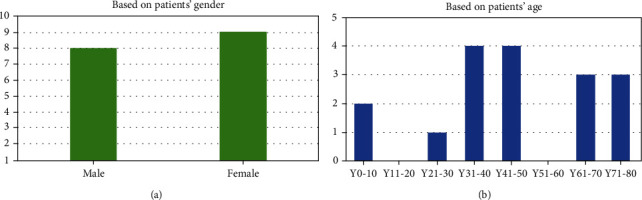
Clinical data of 17 MSK patients (a) based on patient's gender; (b) based on patient's age.

**Figure 3:**
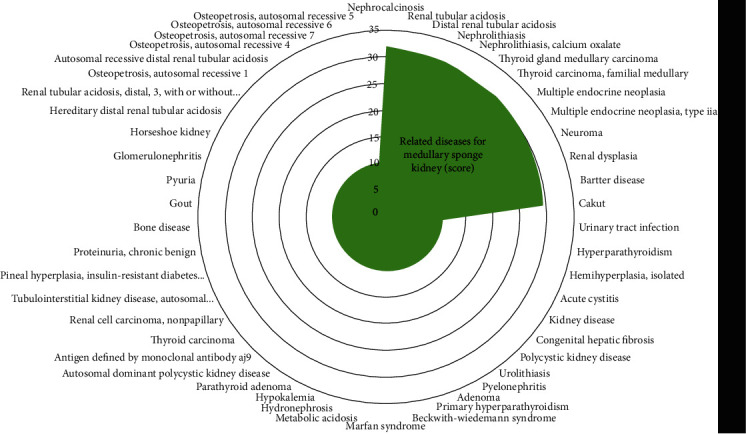
The top 10 related diseases of MSK (MalaCards or GeneCards Suite gene sharing).

**Figure 4:**
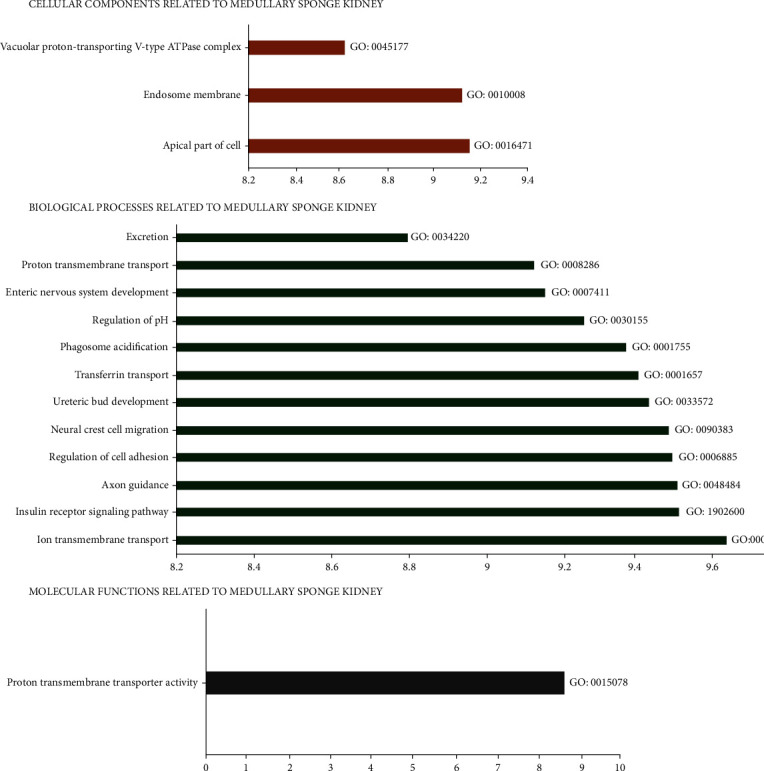
GO analysis (cellular components, biological processes, and molecular functions) of MSK (GeneCards: The Human Gene Database).

17 patients with MSK hospitalized in our hospital were classified according to gender and age by bar chart (Figure 2). We preliminarily found that the incidence of MSK tended to be the same in male and female, and the onset age of MSK was probably 31-50 years old. The top 10 related diseases of MSK via text searches within MalaCards or GeneCards Suite gene sharing were nephrocalcinosis (score: 31.8), renal tubular acidosis (score: 31.2), distal renal tubular acidosis (score: 31.0), nephrolithiasis (score: 30.9), nephrolithiasis, calcium oxalate (score: 30.3), thyroid gland medullary carcinoma (score: 30.3), thyroid carcinoma, familial medullary (score: 30.2), multiple endocrine neoplasia (score: 30.0), multiple endocrine neoplasia, type 2A (score: 29.9), and neuroma (score: 29.7) (Figure 3). In addition, human phenotype analyses of MSK were showed according to the Human Phenotype Ontology (Table 2) and GO analysis of MSK were showed according to GeneCards Suite gene sharing (Figure 4). Cellular components of MSK were GO:0045177 (apical part of cell), GO:0010008 (endosome membrane), and GO:0016471 (vacuolar proton-transporting V-type ATPase complex). Biological processes of MSK were GO:0034220 (ion transmembrane transport), GO:0008286 (insulin receptor signaling pathway), GO:0007411 (axon guidance), GO:0030155 (regulation of cell adhesion), GO:0001755 (neural crest cell migration), GO:0001657 (ureteric bud development), GO:0033572 (transferrin transport), GO:0090383 (phagosome acidification), GO:0006885 (regulation of pH), and GO:0048484 (enteric nervous system development). Molecular function of MSK was GO:0015078 (proton transmembrane transporter activity).

### 3.3. Suspected Genes Related to MSK

**Figure 5:**
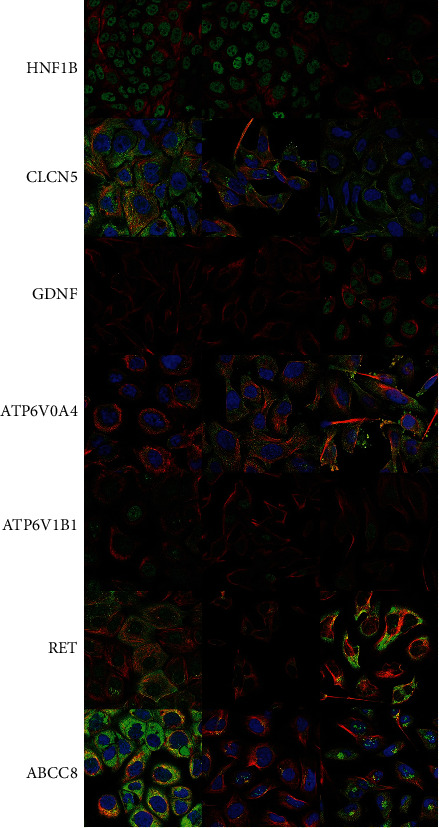
The protein expression location of genes (HNF1B, CLCN5, GDNF, ATP6V0A4, ATP6V1B1, RET, and ABCC8) in human cells.

**Figure 6:**
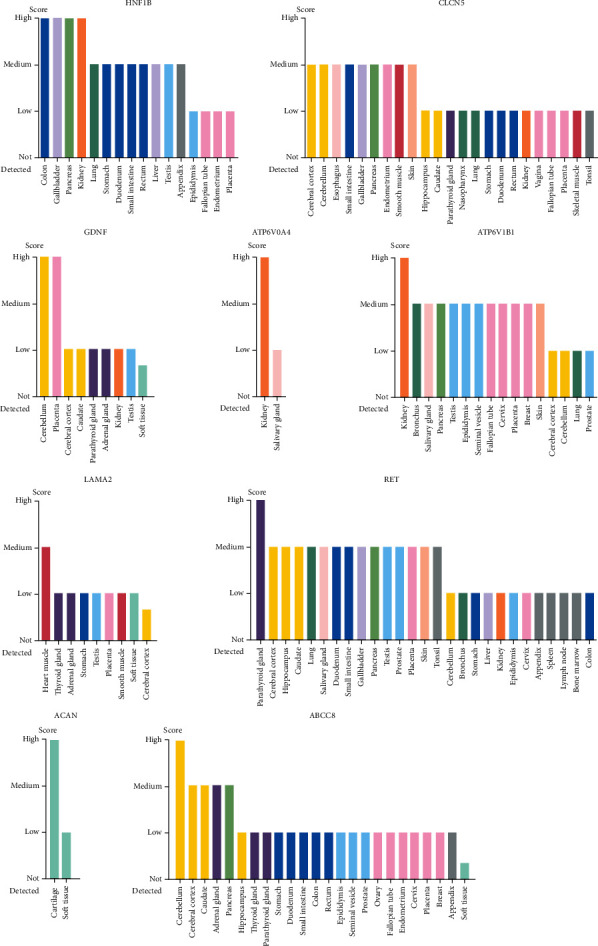
The protein expression of genes (HNF1B, CLCN5, GDNF, ATP6V0A4, ATP6V1B1, RET, and ABCC8) in different tissues.

**Figure 7:**
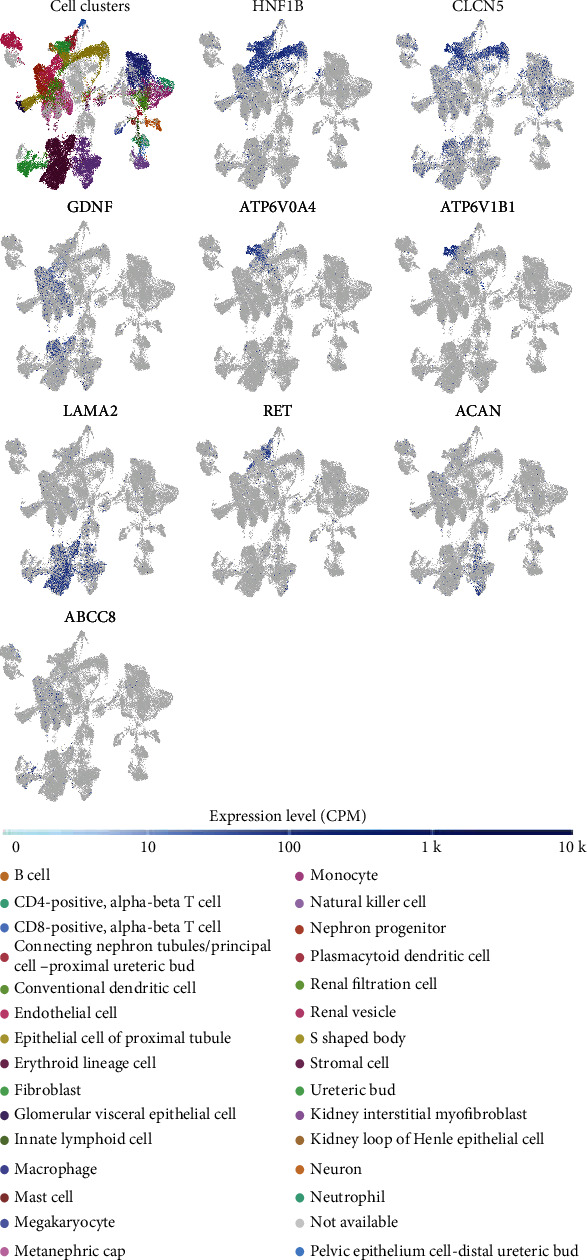
Different gene expression in spatiotemporal immune zonation of the human kidney (single-cell RNA-seq mRNA baseline).

**Figure 8:**
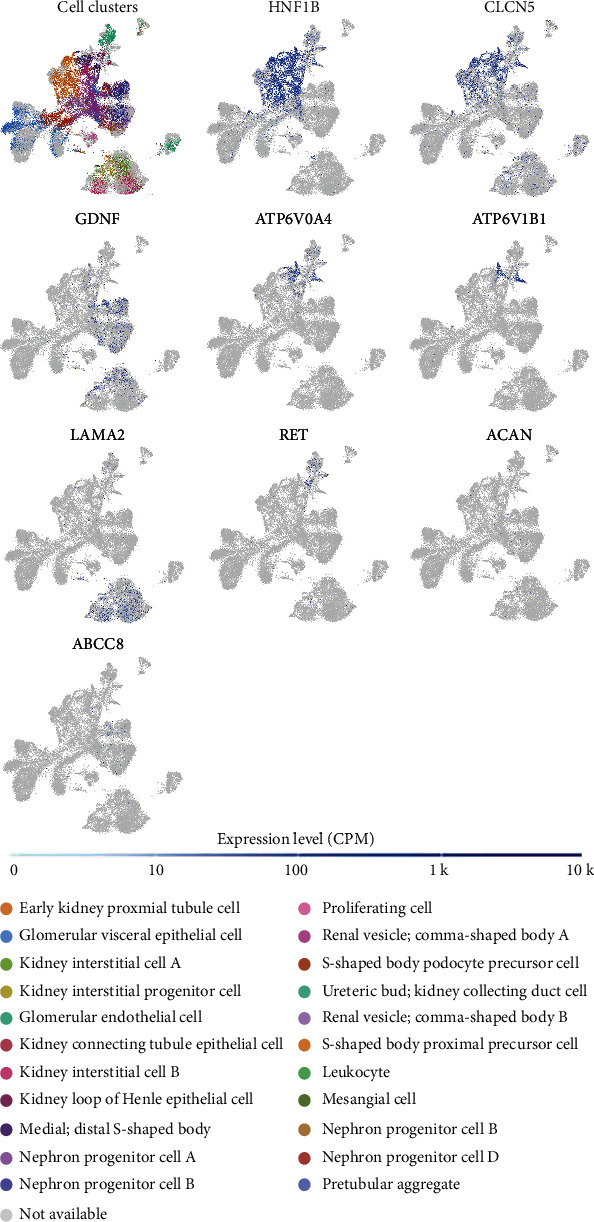
Different gene expression in single cell RNA-sequencing of human fetal kidneys (single-cell RNA-seq mRNA baseline).

There were 7 suspected genes of MSK pathogenesis (Table 3). At present, HNF1B is the only gene that has been confirmed to be associated with the pathogenesis of MSK [7]. In addition, among our 17 patients, a five-year-old girl with MSK underwent genetic testing and was found to have mutations in ACAN and ABCC8 genes, so ACAN, ABCC8, and HNF1B genes were included in the following study (Figures 5 and 6).  Figure 5 shows the protein expression location of genes in human cells. HNF1B is mainly localized to the nucleoplasm and also localized to vesicles. CLCN5 is mainly localized to the plasma membrane and the Golgi apparatus and also localized to the cytosol. GDNF is mainly localized to vesicles and also localized to the nucleoplasm. ATP6V0A4 is localized to the cytosol. ATP6V1B1 is localized to the nucleoplasm and nuclear membrane. RET is mainly localized to the cytosol and the Golgi apparatus and also localized to the plasma membrane. ABCC8 is mainly localized to the nucleoli and also localized to the cytosol and the Golgi apparatus. The protein expression locations of LAMA2 and ACAN were still unknown. The single-cell gene expressions of suspected genes (HNF1B, CLCN5, GDNF, ATP6V0A4, ATP6V1B1, LAMA2, RET, ACAN, and ABCC8) were showed in spatiotemporal immune zonation of the human kidney (Figure 7) and single-cell RNA-sequencing of human fetal kidneys (Figure 8). The result showed that the expression of these genes has been implicated in both human kidney immune zonation and fetal kidney development.

## 4. Discussion

In order to find a better understanding of the heterogeneity of MSK, the molecular basis of this disease has been explored in recent studies. It was reported that GDNF plays an important role in renal development in inducing the growth of the ureteric bud [6]. If the metanephric blastema is not induced by GDNF, the lower part of the nephron cannot continue to grow and differentiate [3]. In addition, an animal study suggested renal hypoplasia had happened in GDNF knockout mice [13]. In other animal studies, variations in the GDNF gene can also cause renal abnormalities, containing kidney atrophy, cortical cysts, and unilateral dysplasia [3]. In addition, mutation of RET can also lead to ureteral bud hypoplasia, renal agenesis, distal nephron dysplasia, and failure of the urinary system to develop [14]. It has been speculated that variation of the RET gene may lead to hyperparathyroidism in MSK patients [15]. However, the role of GDNF and RET in inducing MSK remains questionable due to the small proportion of cases with GDNF or RET mutations. Pathogenic mutations in HFN1B also can cause a variety of renal disorders in patients including tubulointerstitial disease, renal cysts, single kidney, horseshoe kidney, hydronephrosis, diabetes insipidus, and hypomagnesemia due to renal magnesium consumption [16–18]. Izzi et al. found the occurrence of MSK in the family with complete deletion of HNF1B on chromosome 17q12 [7]. Similarly, mutation in CLCN5 can lead the occurrence of renal tubular acidosis, proteinuria, hypercalciuria, renal calcification, kidney stones, and Dent disease [19, 20]. In the kidney, CLCN5 encodes a chloride channel Cl-/H+ exchanger ClC-5, which played an important role in preventing protein loss, and this effect was weakened in patients with Dent disease who carried the defective CLC-5 [21]. Moreover, it was reported that primary distal renal tubular acidosis was a genetic disease caused by the mutation in ATP6V0A4 and ATP6V1B1, which can encode transporters that regulate acid-base balance in collecting tubes [22, 23]. In addition, LAMA2 was reported to be a biomarker for MSK and can be used for early diagnosis of this disorder [24].

In this research, we focused on 17 patients with MSK over the last 10 years, providing some advanced clinical and molecular basis findings of MSK. With respect to the disease phenotype, the advanced findings were twofold.

First, MSK complicated with other diseases in patient 12, patient 13, patient 15, and patient 17 have not been previously reported in existing studies. The most typical complication of MSK was recurrent kidney stones. Pyelonephritis, the second most common complication of MSK, was caused by urinary stasis in the anterior tubules of dilated capillaries, as well as the stones themselves, which can eventually lead to end-stage kidney disease [25], and other clinical phenotypes are also being identified. In our study, MSK concomitant with septic stock, MSK concomitant with ovarian teratoma, MSK concomitant with schwannoma or dermoid cysts, MSK concomitant with gallbladder polyp and MSK concomitant with renal artery stenosis were very rare clinical phenotypes in patients, which were reported for the first time. Based on our clinical findings and existing studies, we concluded that mutations or abnormalities in genes required for normal renal formation may lead to abnormal or reduced nephron development, with the anterior calyceal and collecting ducts primarily affected, leading to cyst formation and ultimately to renal calcification and tubular acidosis. At the same time, our results showed that genes involved in kidney development were also highly expressed in other tissues, which can explain why MSK was often associated with other systemic diseases. Molecular basis research was helpful for the early diagnosis and preventive intervention of MSK patients, and also revealed its possible pathogenesis.

The second important finding was the occurrence of genetic heterogeneity in MSK patients. In patient 9, a female child patient with MSK had concomitant inherited metabolic diseases, low calcium tetany, hypokalemia, hyponatremia, hypocalcemia, malnutrition III, dwarfism, and hypoparathyroidism. Interestingly, we found the mutations of ACAN and ABCC8 in this patient, which was the first reported in the world. ACAN variants have been reported to be associated with short stature in recent researches [26, 27]. Additionally, ABCC8 was thought to be involved in the development of diabetes [28, 29]. However, there was no direct evidence that ACAN and ABCC8 mutations were associated with the incidence of MSK. In patient 4, a pregnant woman with MSK complicated with double renal calculi, type 2 diabetes, and hypokalemia and has a baby with neonatal hypospadias. As not all patients with MSK have a family history of the disease [30], a genetic variant form of MSK were possible. MSK was often associated with renal or urinary tract abnormalities. In addition, the expression of HNF1B, CLCN5, GDNF, ATP6V0A4, ATP6V1B1, LAMA2, RET, ACAN, and ABCC8 have been implicated in both human kidney immune zonation and fetal kidney development, suggesting the existence of developmental abnormalities. In combination with our clinical cases and recent studies, we suspect that MSK is more likely to be a manifestation of renal dysplasia caused by changes in some key developmental genes that do not absolutely manifest as MSK in the course of inheritance, which may also cause other developmental abnormalities in the urinary system. At present, there is no definite and effective treatments for MSK. The goal of almost all treatments is to maintain renal function as much as possible. Perhaps by tracking the changes in gene expression, it is promising to evaluate the effect of treatments or disease progression.

## 5. Conclusion

To sum up, we concluded that NF1B, CLCN5, GDNF, ATP6V0A4, ATP6V1B1, LAMA2, RET, ACAN, and ABCC8 could be independent indicators for the diagnosis and preventive intervention of MSK patients, and abnormal kidney development due to mutations in key genes was the underlying cause of MSK.

## Figures and Tables

**Table 1:** Clinical data of 17 patients with medullary sponge kidney.

No.	Age	Gender	Primary diagnosis	Secondary diagnosis	Supplement
Patient 1	Y49	Female	Medullary sponge kidney	(1) Double renal calculi, (2) left upper ureteral calculi, (3) left uronephrosis, (4) renal insufficiency, (5) urinary infection, (6) hypokalemia, (7) hypoproteinemia, (8) anemia	
Patient 2	Y66	Male	Medullary sponge kidney	(1) Double renal calculi, (2) urethrostenosis, (3) renal insufficiency, (4) urinary infection, (5) hyperthyroidism, (6) metabolic acidosis, (7) hypertension, (8) anemia	
Patient 3	Y74	Male	Medullary sponge kidney	(1) Double renal calculi, (2) double renal cyst, (3) renal insufficiency, (4) ileus, (5) metabolic acidosis, (6) hyperkalemia, (7) hyponatremia, (8) hypochloremia, (9) anemia	
Patient 4	Y33	Female	Medullary sponge kidney	(1) Double renal calculi, (2) G2P2 39 W +3 LOA, (3) single live birth–male, (4) type 2 diabetes, (5) hypokalemia	Neonatal hypospadias
Patient 5	Y73	Female	Medullary sponge kidney	(1) Left renal calculi, (2) double renal abscess, (3) hypokalemia, (4) hyponatremia, (5) hypochloremia	
Patient 6	Y44	Female	Medullary sponge kidney	(1) Right renal calculi, (2) Hashimoto's thyroiditis, (3) chronic superficial gastritis, (4) dyslipidemia, (5) polypofgallbladder	
Patient 7	Y71	Male	Medullary sponge kidney	(1) Left renal calculi, (2) left renal abscess, (3) right pelvic effusion, (4) right spontaneous pneumothorax, (5) liver cyst, (6) gallstone	
Patient 8	Y42	Male	Medullary sponge kidney	(1) Double renal calculi, (2) double renal cyst, (3) greater occipital neuralgia, (4) hypertension	
Patient 9	Y5	Female	Medullary sponge kidney	(1) Inherited metabolic diseases, (2) low calcium tetany, (3) hypokalemia, (4) hyponatremia, (5) hypocalcemia, (6) malnutrition iii, (7) dwarfism, (8) hypoparathyroidism	Mutations in ACAN, ABCC8
Patient 10	Y32	Female	Medullary sponge kidney	(1) Double renal calculi, (2) left upper ureteral calculi	
Patient 11	Y46	Male	Medullary sponge kidney	(1) Double renal cyst, (2) left uronephrosis, (3) hypokalemia, (4) hypertension, (5) pituitary microadenoma	
Patient 12	Y69	Female	Medullary sponge kidney	(1) Septic stock, (2) double renal calculi, (3) left renal cyst, (3) right ureteral calculi, (4) renal insufficiency, (5) urinary infection, (6) anemia	
Patient 13	Y27	Female	Medullary sponge kidney	(1) Left renal calculi, (2) right ovarian teratoma, (3) left breast fibroadenoma, (4) breast hyperplasia	
Patient 14	Y2	Male	Medullary sponge kidney	(1) Double renal calculi, (2) bladder calculi, (3) uroschesis	
Patient 15	Y39	Male	Medullary sponge kidney	(1) Left forearm schwannoma, (2) multiple dermoid cysts, (3) gallbladder polyp	
Patient 16	Y63	Male	Medullary sponge kidney	(1) Double renal calculi, (2) left renal cyst	
Patient 17	Y33	Female	Medullary sponge kidney	(1) Left renal calculi, (2) left renal artery stenosis, (3) hypertension, (4) hyperuricemia, (5) dyslipidemia	

**Table 2:** Human phenotypes related to medullary sponge kidney.

Description	HPO frequency	Orphanet frequency	HPO source accession
Nephrolithiasis	Hallmark (90%)	Very frequent (80-99%)	HP:0000787
Hematuria	Frequent (33%)	Frequent (30-79%)	HP:0000790
Hypercalciuria	Frequent (33%)	Frequent (30-79%)	HP:0002150
Distal renal tubular acidosis	Frequent (33%)	Frequent (30-79%)	HP:0008341
Hemihypertrophy	Occasional (7.5%)	Occasional (5-29%)	HP:0001528

**Table 3:** Genes related to medullary sponge kidney.

Suspected gene	Description	Evidence
HNF1B	HNF1 Homeobox B	Causative germline mutation [7]
CLCN5	Chloride voltage-gated channel 5	Inferred via ClinVar and dbSNP
GDNF	Glial cell derived neurotrophic factor	Inferred via GeneCards
ATP6V0A4	ATPase H+ transporting V0 subunit A4	Inferred via GeneCards
ATP6V1B1	ATPase H+ transporting V1 subunit B1	Inferred via GeneCards
LAMA2	Laminin subunit alpha 2	Inferred via GeneCards
RET	Ret protooncogene	Inferred via GeneCards

## Data Availability

The data used to support the findings of this study are available from the corresponding author upon request.
